# Incorporating 16S Gene Copy Number Information Improves Estimates of Microbial Diversity and Abundance

**DOI:** 10.1371/journal.pcbi.1002743

**Published:** 2012-10-25

**Authors:** Steven W. Kembel, Martin Wu, Jonathan A. Eisen, Jessica L. Green

**Affiliations:** 1 Institute of Ecology & Evolution, University of Oregon, Eugene, Oregon, United States of America; 2 Department of Biology, University of Virginia, Charlottesville, Virginia, United States of America; 3 UC Davis Genome Center, University of California Davis, Davis, California, United States of America; 4 Department of Evolution and Ecology, College of Biological Sciences, University of California Davis, Davis, California, United States of America; 5 Department of Medical Microbiology and Immunology, School of Medicine, University of California Davis, Davis, California, United States of America; 6 Santa Fe Institute, Santa Fe, New Mexico, United States of America; University of Zurich and Swiss Institute of Bioinformatics, Switzerland

## Abstract

The abundance of different SSU rRNA (“16S”) gene sequences in environmental samples is widely used in studies of microbial ecology as a measure of microbial community structure and diversity. However, the genomic copy number of the 16S gene varies greatly – from one in many species to up to 15 in some bacteria and to hundreds in some microbial eukaryotes. As a result of this variation the relative abundance of 16S genes in environmental samples can be attributed both to variation in the relative abundance of different organisms, and to variation in genomic 16S copy number among those organisms. Despite this fact, many studies assume that the abundance of 16S gene sequences is a surrogate measure of the relative abundance of the organisms containing those sequences. Here we present a method that uses data on sequences and genomic copy number of 16S genes along with phylogenetic placement and ancestral state estimation to estimate organismal abundances from environmental DNA sequence data. We use theory and simulations to demonstrate that 16S genomic copy number can be accurately estimated from the short reads typically obtained from high-throughput environmental sequencing of the 16S gene, and that organismal abundances in microbial communities are more strongly correlated with estimated abundances obtained from our method than with gene abundances. We re-analyze several published empirical data sets and demonstrate that the use of gene abundance versus estimated organismal abundance can lead to different inferences about community diversity and structure and the identity of the dominant taxa in microbial communities. Our approach will allow microbial ecologists to make more accurate inferences about microbial diversity and abundance based on 16S sequence data.

## Author Summary

Microbial ecologists cannot observe their study organisms directly, so they use molecular sequencing to measure the abundance of different microbes living in the wild. The most commonly used method for measuring the abundance of different microbes is to collect a DNA sample from an environment and sequence a particular gene, the 16S SSU rRNA gene (“16S”) from those samples. The abundance of 16S sequences from different microbes is then used as a surrogate measure of the abundance of the microbial taxa in the community. One problem with the use of the 16S gene as a measure of microbial abundance is that many microbes have multiple copies of the gene in their genome. Thus, variation in 16S gene abundances can be caused by both genomic copy number variation and variation in the abundance of organisms. In this study we present a computational method that allows estimation of the abundance and genomic 16S copy number of microbes based on environmental sequencing of the 16S gene. We use simulations and analysis of microbial community data sets to demonstrate that estimating the abundance of organisms from 16S data improves our ability to accurately measure the diversity and abundance of microbial communities.

## Introduction

The SSU rRNA gene (also known as the 16S rRNA gene, referred to as “16S” hereafter) is widely used in studies of microbial ecology as a “barcode gene” [1] to quantify microbial community structure and diversity [2], [3]. The widespread adoption of 16S as a microbial barcode gene has been driven by several desirable properties of the gene, including the fact that it is universal across bacteria and archaea, it can be easily amplified from a wide diversity of taxa at one time by the polymerase chain reaction (PCR), it is phylogenetically informative, and it can be used to identify and phylotype sequences based on extensive databases of 16S sequences with associated taxonomic and phylogenetic information [2], [4]. In 2011 there were 3,574 publications in the Web of Science database matching a search for the terms “16S and (communit* or diversit* or abundance*)”.

**Figure 1:**
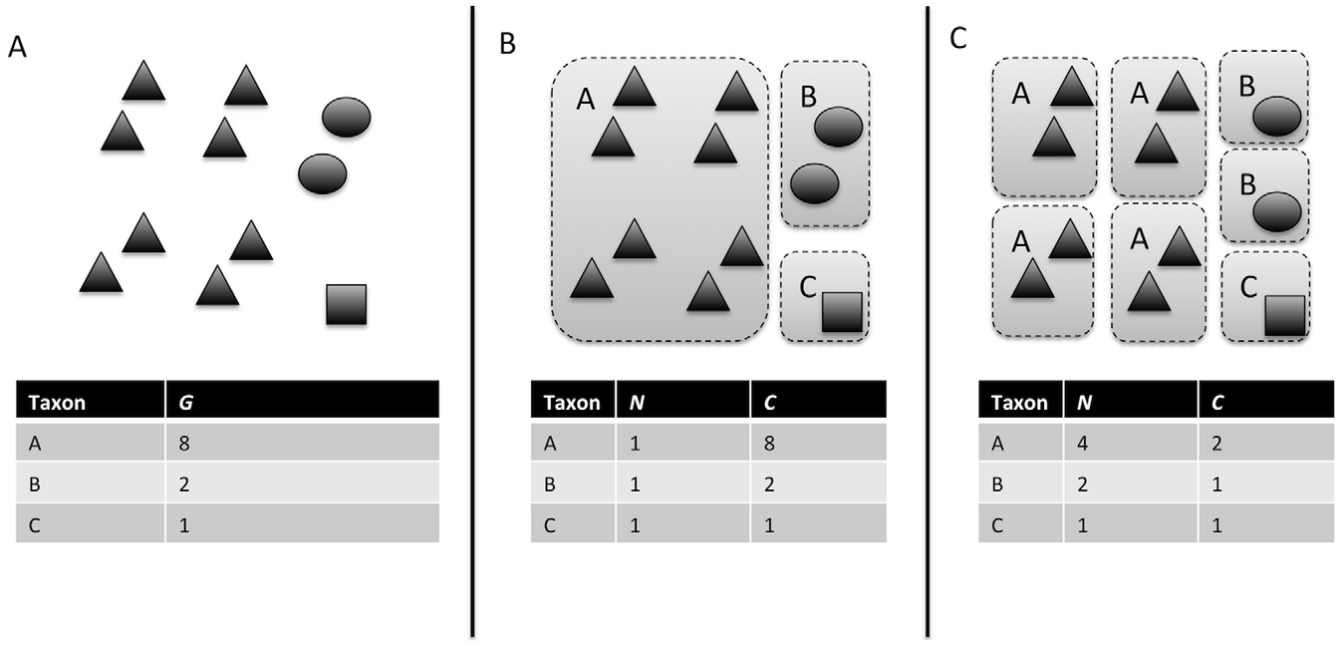
Conceptual diagram illustrating how variation in genomic 16S copy number could influence observed abundance of 16S gene sequences in a community. Observed 16S gene sequence abundances (*G*) in an environmental sequencing data set (**A**) could be generated by a variety of underlying organismal abundance distributions (*N*; e.g. **B** or **C**) depending on the genomic copy number of the 16S gene (*C*) within individual cells of the organisms in the community (gray rectangles denote single cells, black symbols denote copies of the 16S gene from different organisms).

There are numerous advantages to using 16S as a microbial community barcode gene, but also numerous disadvantages including amplification and sequencing bias and error [5], [6], difficulty with the accurate taxonomic identification and binning of short sequences [7]–[10], and a lack of benchmark studies to guide decisions about quality control, filtering, and analysis of 16S sequence data sets derived from novel sequencing technologies. Another disadvantage of the 16S gene that is particularly relevant to inferring microbial abundance from 16S gene sequence abundances is that genomic 16S copy number varies a great deal across the tree of life [11]–[13]. For example, among bacterial taxa with fully sequenced genomes, 16S copy number varies from a single copy in *Erythrobacter litoralis* to fifteen copies in *Photobacterium profundum* [14], [15]. As a result of this variation in copy number, the variation in the relative abundance of 16S gene sequences in an environmental sample can be attributed both to variation in the relative abundance of different organisms, and to variation in genomic 16S copy number among those organisms ([12], [16]–[18]; Figure 1). The use of a single-copy protein coding gene such as *rpoB* as a microbial barcode would avoid this problem [11], [19], [20], but these genes are not as widely used as the 16S gene, and there are biases inherent in the use of every barcode gene and sequencing technology. Though metagenomic data will help in allowing the use of genes that have less variation in copy number [20], PCR amplification of 16S genes is still the method of choice in many environmental surveys. The vast majority of such studies either explicitly or (usually) implicitly assume that the relative abundance of 16S gene sequences is an accurate measure of relative abundance of the organisms containing those sequences in analyses of community diversity and composition. The degree to which this assumption is warranted, and the effect of treating 16S gene abundance as a surrogate measure of organismal abundance on estimates of microbial community structure, is unknown.

In this study we present a method for phylogenetic estimation of 16S copy number and organismal abundance that allows us to improve estimation of microbial abundance and community structure by accounting for copy number variation among taxa. Our specific objectives are threefold. First, we demonstrate that 16S gene abundance is a function of both organismal abundance and 16S gene copy number, and show how this relationship can influence the ability to estimate community structure and diversity from sequence data. Second, we develop a method that allows estimation of organismal 16S gene copy number and abundance as a function of 16S gene abundances in environmental samples, and assess the performance of this method with simulated data sets. Finally, we apply our method to several empirical data sets to illustrate the practical effects of treating 16S gene abundance as a measure of organismal abundance on measures of microbial community diversity, structure, and composition.

## Methods

### Linking 16S gene abundance, copy number, and organismal abundance

Our interest lies in relating the observed abundances of 16S genes in biological samples to the abundance of cells, or organisms, from which these genes arise. For any taxon *i* within a biological community, the relationship between the abundance of 16S genes from that taxon, *G_i_*, and the abundance of organisms from that taxon, *N_i_*, is determined by the genomic 16S copy number of that taxon, *C_i_*, where *G*_i_ = *N_i_C_i_*. Defining the relative 16S gene abundance of taxon *i*, 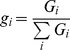, and the relative organismal abundance of taxon *i*, 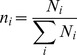, it follows that:
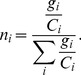
(1)Here, the summation 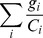 is across all taxa *i* within the biological community, and is thus a constant. Because 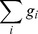 = 1, equation 1 shows that in communities where all taxa have 16S copy number equal to one, all sampled taxa will have identical 16S gene and organismal relative abundance. As the 16S copy number of one or more taxa increases, disparity between the 16S gene abundance and organismal abundance of individual taxa grows.

We can also readily explore community-level patterns of microbial abundance. We characterize the taxa-gene distribution, *P*(*G*), as the fraction of taxa in a biological sample with 16S gene abundance *G*. Similarly, we characterize the taxa-abundance distribution, *P*(*N*), as the fraction of taxa with *N* organisms. These two distributions are related by:
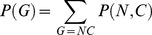
(2)Here, the summation is over all possible combinations of *N* and *C* with product equal to *G*, and *P*(*N*,*C*) is the joint probability of a taxon having an abundance *N* and copy number *C*. In the case where organismal abundance and copy number are independent of one another, this simplifies to:
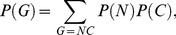
(3)where *P*(*C*) is the distribution of copy number across taxa within the biological community.

To understand the potential differences between gene abundance distributions and the organismal abundance distributions from which they are derived, we used two approaches. First we qualitatively compared the shapes of the distributions of *P*(*N*) and *P*(*G*). To model the taxa-abundance distribution, *P*(*N*), we simulated biological communities assuming a zero-truncated Poisson lognormal distribution [21]. We chose the lognormal distribution for illustrative purposes because it is one of the most widely discussed taxa-abundance distributions in biology [22], [23]. To model the distribution of genomic 16S copy number across taxa, *P*(*C*), we simulated biological communities with a zero-truncated Poisson distribution. We chose the Poisson distribution because it approximated the empirical copy number distribution in our reference data set (Supporting Figure S1). For each simulated community we calculated the resulting taxa-gene distribution, *P*(*G*), from equation 3.

Second, we examined how sampling from the simulated biological communities with corresponding distributions *P*(*N*) and *P*(*G*) resulted in different biodiversity estimates. Our motivation for this was to understand the differences expected when sampling genes versus individuals from communities. To do this we sampled a fixed number of genes, or individuals, from the simulated communities. We focused on a key attribute of the sample distributions: the numbers of taxa that are unobserved, or hidden behind the ‘veil line’ of the sampled taxa-abundance and taxa-gene distributions [22]. For each sample we used standard parametric tools to estimate the number of unobserved taxa for *P*(*N*) and *P*(*G*) (reviewed in [24]). We tested whether estimating the total taxa richness based on *P*(*G*) versus *P*(*N*) could lead to different inferences about diversity using an ANOVA to compare predicted taxa richness using these two different distributions.

### Estimating copy number and organismal abundance using 16S gene sequences

Environmental sequencing studies that utilize the 16S gene as a barcode provide a measure of 16S gene relative abundance *g_i_*. Given the relationship between 16S gene relative abundance *g_i_*, copy number *C_i_*, and organismal relative abundance *n_i_* outlined above (Equation 1), we can estimate *n_i_* given information on *g_i_* and *C_i_*. But the genomic copy number *C_i_* of the 16S gene (referred to as “copy number” hereafter) is usually not observed directly from environmental sequence data because the full genomes of the organisms containing the gene are not sequenced. Metagenomic studies could theoretically address this issue [19], , but metagenomic sequencing generally provides insufficient sampling depth to provide full genome coverage for all of the organisms in diverse communities. To overcome this challenge, we use methods from comparative biology and leverage phylogenetic signal in copy number to estimate copy number and organismal abundance for organisms for which we observe only 16S gene abundances.

**Figure 2:**
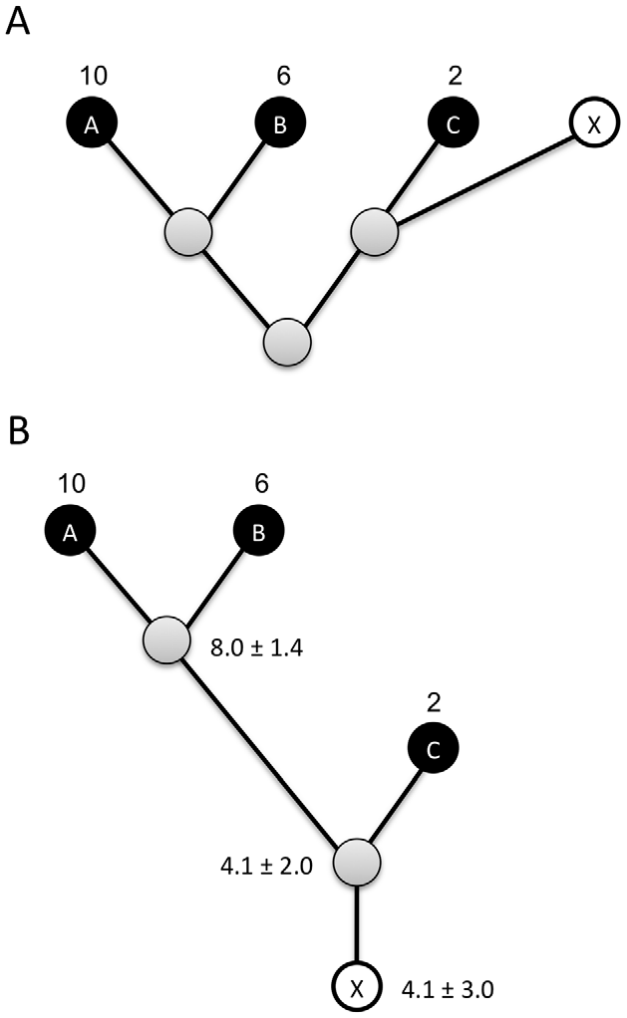
Conceptual diagram showing how copy number can be estimated for environmental sequences using a reference phylogeny. Given a reference phylogeny with copy number known for species A, B, and C, trait values for a hypothetical novel taxon or sequence X (**A**) can be estimated in a phylogenetically independent contrasts framework by rerooting the phylogeny at the ancestor of X and its closest relative in the reference phylogeny (**B**). After rerooting, a predicted trait value and standard error for X can be calculated using ancestral state reconstruction.

The general approach we use to estimate copy number and organismal abundance from environmental 16S sequences is to place those sequences onto a reference phylogeny of organisms for which genomic 16S copy number is known (Figure 2). Using ancestral state reconstruction via phylogenetically independent contrasts [26], [27], we can then obtain an estimate of genomic 16S gene copy number, 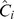, for any taxon *i*. By combining the estimated copy number 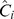, and the observed relative gene abundance of taxon *i*, *g_i_*, we can obtain an estimate of the relative abundance of taxon *i* following Equation 1:
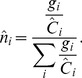
(4)


#### Reference database construction

We created a reference database of taxa for which full-length 16S gene sequences and estimates of genomic 16S gene copy number were available, based on a data set of 881 bacterial taxa with fully sequenced genomes [28]. Reference sequences were aligned to the GreenGenes core set [29] with PyNAST [30] and masked with the GreenGenes lanemask. We constructed a phylogeny of the reference sequences using RAxML [31] with a GTR+Gamma model of evolution.

The reference data set contained several clades of very closely related taxa (i.e. 27 strains of *Escherichia coli*). Because short read placement methods and ancestral state reconstruction methods do not deal well with the extremely short or zero-length branches linking taxa in these clades, we pruned the reference data set so that for groups of very closely related organisms separated by branch lengths <0.01, a single representative of the group was chosen at random and retained and the others were eliminated. This resulted in the elimination of 397 of the 881 reference taxa, leaving 484 taxa in the pruned reference data set. The eliminated taxa were almost exclusively multiple strains of a single bacterial species, or multiple species from a genus, with similar or identical genomic 16S copy number.

Measures of 16S copy number for each reference taxon were obtained through enumeration of genes annotated as 16S rRNA genes in each reference taxon's genome. For genomes for which the 16S rRNA genes were not annotated, the RNAmmer program was used to identify the 16S rRNA genes [32]. We assessed the accuracy of our method for estimating genomic 16S copy number by comparing our estimates of copy number with estimates for the same strains in another database of genomic 16S copy number estimates (the rrnDB database [15]). For the 521 strains present in both data sets, copy number estimates were almost perfectly correlated (r = 0.99, P<0.001) and agreed exactly or differed by a single copy for 99% of strains.

The use of phylogenetic methods for copy number estimation depends on the existence of a phylogenetic signal in genomic 16S copy number. Phylogenetic signal is a tendency for closely related species to possess similar values of a trait [33]. We measured phylogenetic signal in 16S copy number using the *K* statistic [34], which compares the amount of signal in a trait to the amount expected under a Brownian motion model of trait evolution. In this framework, higher values of *K* indicate stronger phylogenetic signal; *K* values close to zero indicate random phylogenetic signal, while *K* = 1 is the expected signal under a Brownian motion model of trait evolution. An associated *P*-value is computed by comparing the variance of phylogenetically independent contrasts for the observed phylogeny and data to the random values expected after permuting taxa labels across the phylogeny. To better meet assumptions of normal distribution, we square-root transformed copy number for all subsequent analyses.

#### Estimating copy number and organismal abundance for novel taxa

We used the framework of phylogenetically independent contrasts [27] to estimate copy number for novel taxa, such as the taxa observed during sequencing of environmental samples. Given a reference phylogeny with copy number known for all reference taxa, to estimate copy number for a novel taxon we reroot the phylogenetic tree at the common ancestor of the novel taxon and its closest relative on the reference phylogeny (Figure 2). Using phylogenetically independent contrasts for ancestral state reconstruction [35], we then estimate the predicted copy number at the new root node of the phylogeny, and use the branch length connecting the root and the novel taxon to adjust our estimate of uncertainty in the novel taxon's 16S copy number. This results in an estimate of 16S copy number plus the uncertainty in that estimate for any taxon that can be placed on the reference phylogeny. Estimated copy number and gene abundance can then be combined following equation 4 to provide an estimate of the relative abundance of the organisms contributing each sequence to the community.

#### Software implementation

We developed a software pipeline to estimate genomic 16S copy number and organismal abundance for 16S sequences derived from an environmental sample. A set of 16S sequences derived from an environmental sample can be aligned and masked to the same Greengenes core data set as the reference taxa using PyNAST [30] as implemented in the QIIME pipeline [36]. The aligned and masked environmental sequences can then be placed on the reference phylogeny using pplacer [37]. The resulting phylogeny of reference plus environmental sequences are then combined with the copy number information to provide estimates of copy number for the environmental sequences, based on phylogenetically independent contrasts ancestral state reconstruction. These copy number estimates can then be used to estimate organismal relative abundance for a community data set of 16S gene abundances. The steps of this pipeline are implemented in a series of command line and R scripts [38] and associated reference data sets (Supporting Dataset S1). Functions for estimation of copy number and organismal abundance will be included in the picante R package [39] and pplacer [37]. Estimation of copy number and organismal abundance for several empirical data sets took approximately one hour per 10,000 sequences on a 2.26 GHz Intel Xeon processor. Thus, the method we present is currently usable with data sets comprised of tens to hundreds of thousands of operational taxonomic units (OTUs), and could easily be extended for use with data sets orders of magnitude larger, since compute time will scale linearly with the number of query sequences, and the algorithm could easily be parallelized.

#### Assessing effects of read length and phylogenetic placement uncertainty on copy number and relative abundance estimation accuracy

We used leave-one-out cross-validation analysis to assess the accuracy of copy number estimates for the 484 reference taxa data set. We measured estimation bias as the mean difference between observed and predicted copy number, and estimation error as the mean absolute difference between observed and predicted copy number. We also assessed the effect of reference data set size on estimation error and bias (see Supporting Text S1 and Supporting Figure S2).

We quantified the performance of our copy number estimation method in terms of ability to estimate organismal abundance accurately, taking into account uncertainty in phylogenetic placement and copy number estimation for environmental sequences. We simulated communities by selecting 100 sequences at random from the pruned reference phylogeny to represent members of an ecological community (OTUs). We simulated organismal relative abundance (*n_i_*) values for each OTU assuming a lognormal distribution of abundance within the community, and calculated *g_i_* (16S gene relative abundance) as a function of *n_i_* and the known genomic 16S copy number *C_i_* for each OTU (Equation 3). We then simulated 16S rRNA sequencing of the simulated community by sampling 1,000 sequences with replacement from the community, with probability of sampling proportional to gene abundance *g_i_*.

For each simulated environmental sample, we placed OTUs from the simulated environmental sample onto a version of the reference phylogeny with the 100 OTUs in the simulated community removed from the reference phylogeny. We then estimated copy number *C_i_* for the 100 OTUs in the community and estimated 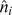. We repeated this simulation 100 times using both the full-length reference sequences, as well as sequences shortened to a length of 351 nucleotides to simulate the read length obtained from high-throughput pyrosequencing of the V2V3 hypervariable region of the 16S gene [40].

These simulations allowed us to evaluate the impact of errors in phylogenetic placement and copy number estimation on the ability of our method to accurately estimate the copy number and relative abundance of taxa from their gene sequences and abundances. We compared the correlations between 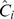 and *C_i_* for the OTUs in each simulated community, and compared the correlations between *g_i_* and 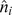 versus *n_i_*.

### Case studies: effect of copy number variation on community structure in empirical data sets

To illustrate the impact of variation in copy number on empirical estimates of microbial community structure and diversity, we reanalyzed data from two previously published studies: a survey of microbial communities along an oceanic depth gradient using Sanger sequencing [41], and a survey of the skin, gut, and mouth microbiome of a human female using pyrosequencing (subject F1-3 from [42]).

For each data set, we estimated the relative abundance 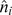 for each OTU using our copy number estimation pipeline. We then asked whether accounting for copy number variation influenced several commonly used measures of community structure and diversity for each data set. We estimated the fit of *g_i_* and 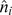 abundance distributions from these data sets to a lognormal model of relative abundance distributions. We classified each sequence in the empirical data sets to the taxonomic order level using the RDP taxonomic classifier [43] and evaluated changes in the relative abundance of bacterial orders based on *g_i_* versus 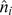. We measured overall community dissimilarity among samples from each study using the weighted UniFrac phylogenetic distance metric [44], based on the both the *g_i_* and 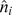 values, and then performed a hierarchical clustering with complete linkage to evaluate the overall similarity of samples in each study.

## Results

### Linking 16S gene abundance, copy number, and organismal abundance

**Figure 3:**
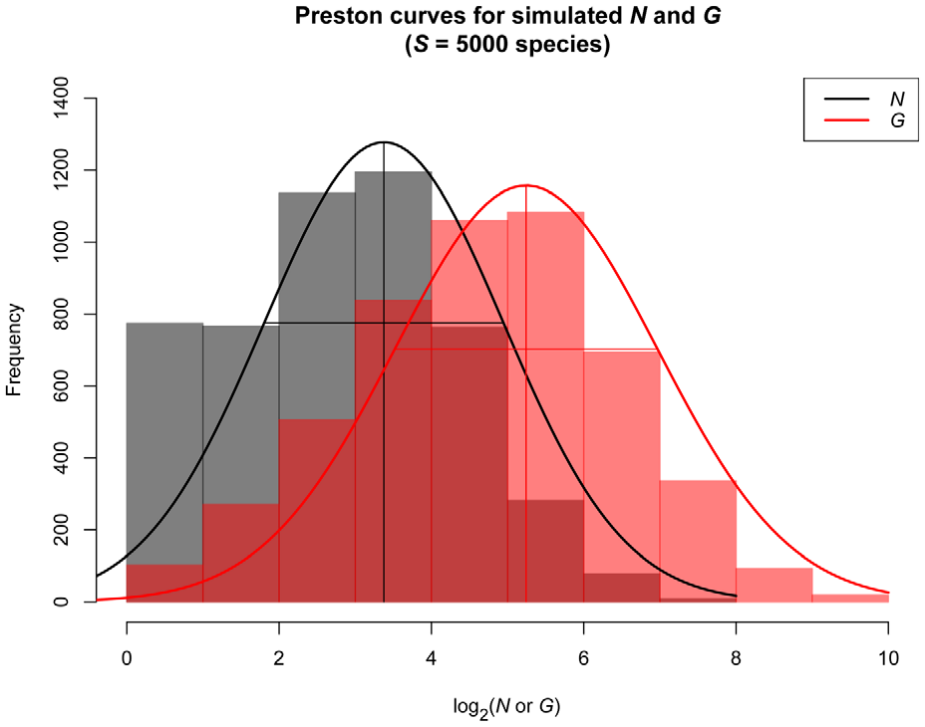
Taxa-abundance and taxa-gene curves (number of species in log_2_-abundance octaves) fit to a simulated distribution of organismal abundances (*N_i_*; black) and resulting gene abundances (*G_i_*; red) for 5000 species. For each species, abundance *P(N)* was simulated as a zero-truncated lognormal distribution (mean = 2, variance = 4), copy number *P(C)* was simulated as a zero-truncated Poisson distribution (mean = 4, variance = 4), and *P(G)* was calculated as *P(G) = P(N)P(C)* following Equation 3.

**Figure 4:**
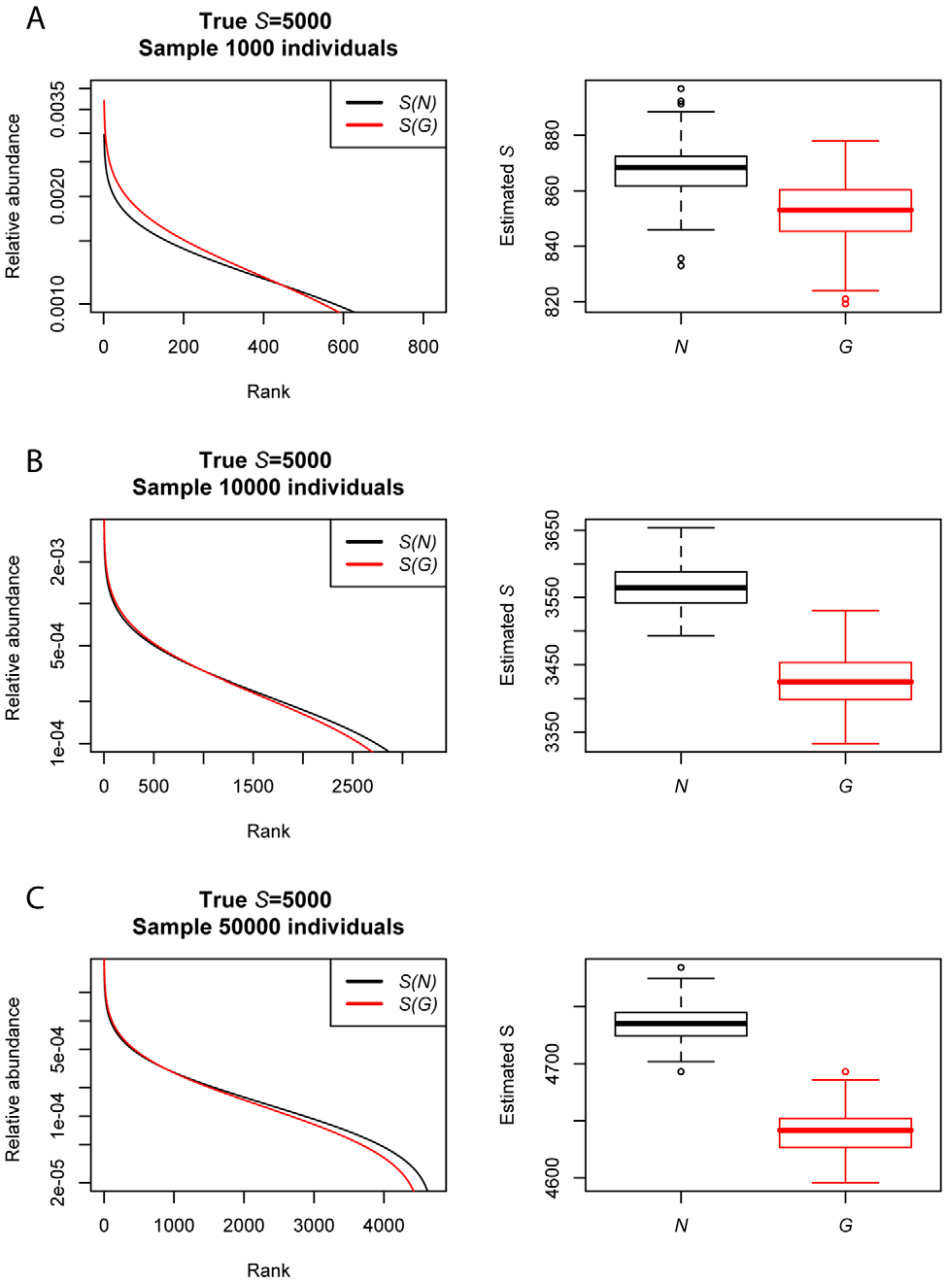
Rank abundance distributions and estimated species pool richness from 100 simulations of communities of (A) 1000, (B) 10000, and (C) 50000 individual genes or organisms sampled from an underlying distribution of abundances (*P(N)*) and genes (*P(G)*). For each simulation, a distribution of organismal abundances (*P(N)*; black) and resulting gene abundances (*P(G)*; red) was generated for 5000 species following the methods described in the caption for Figure 3. Rank-abundance distributions are presented for a single randomly chosen simulation at each sampling intensity. For each simulation, we estimated the number of species *S* in the species pool using a parametric method [22], [23], with the true *S* = 5000. Estimates of species pool size were significantly higher and closer to the true value based on *N* versus *G* at all sampling intensities (ANOVA; P<0.01).

Plots of simulated *P*(*N*) and *P*(*G*) abundance distributions (Figure 3) indicated that the shape of these distributions are different. For the simulation parameters we considered, treating *G_i_* as a measure of organismal abundance lead to an underestimation of the abundance of rare taxa and overestimation of the abundance of the most abundant taxa compared to the distribution of *N_i_* (Figures 3 and 4). Estimates of total species pool richness fit using a parametric method [23] were significantly lower for *G_i_* than for *N_i_* (ANOVA; all *P*<0.01; Figure 4).

### Estimating copy number and organismal abundance using 16S gene sequences

#### Phylogenetic signal in copy number and copy number estimation accuracy

**Figure 5:**
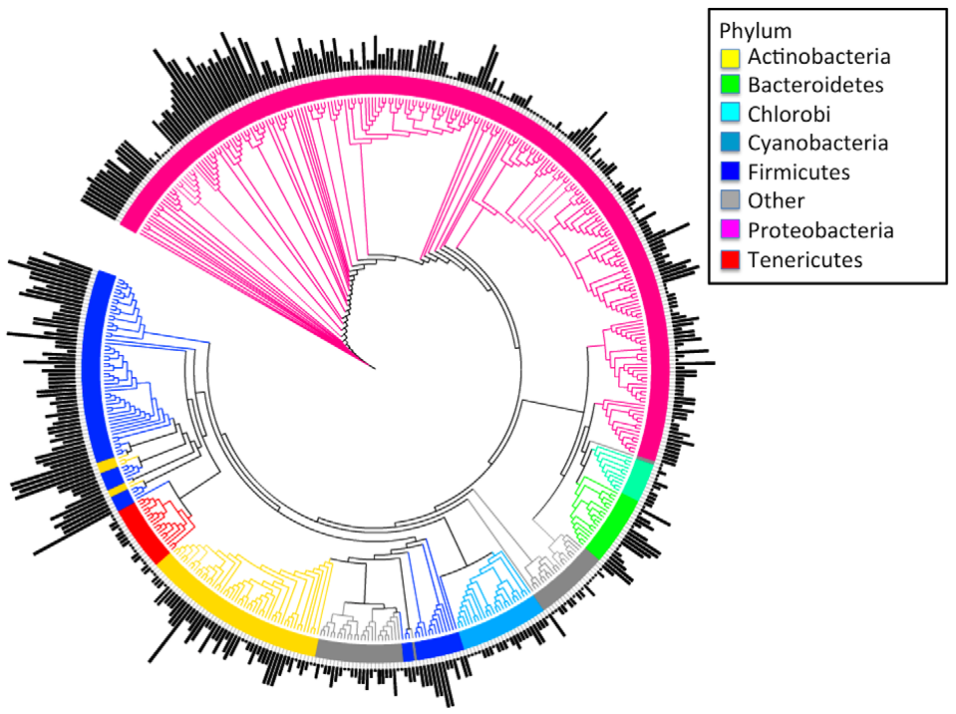
Bacterial reference phylogeny with genomic 16S copy number indicated with black bars (bar length proportional to genomic 16S copy number) and taxonomic order (determined using RDP Taxonomic Classifier [**43**]) indicated with color shading of branches.

There is phylogenetic signal in 16S copy number in bacteria (Figure 5; *K* = 0.48, *P*<0.001) based on analysis of square-root transformed copy number for the 484 reference taxa, supporting the use of a phylogenetic approach to predicting copy number. The leave-one-out cross-validation analysis of observed and predicted copy number for the 484 reference taxa indicated that copy number can be predicted accurately through the use of phylogenetic prediction methods; the mean prediction bias (the mean difference between observed and predicted copy number) for the 484 reference taxon data set was −0.05 copies and the mean prediction error (the mean absolute difference between observed and predicted copy number) was 1.04 copies (Supporting Text S1, Supporting Figure S2).

#### Effects of read length and phylogenetic placement uncertainty on relative abundance estimation accuracy

**Figure 6:**
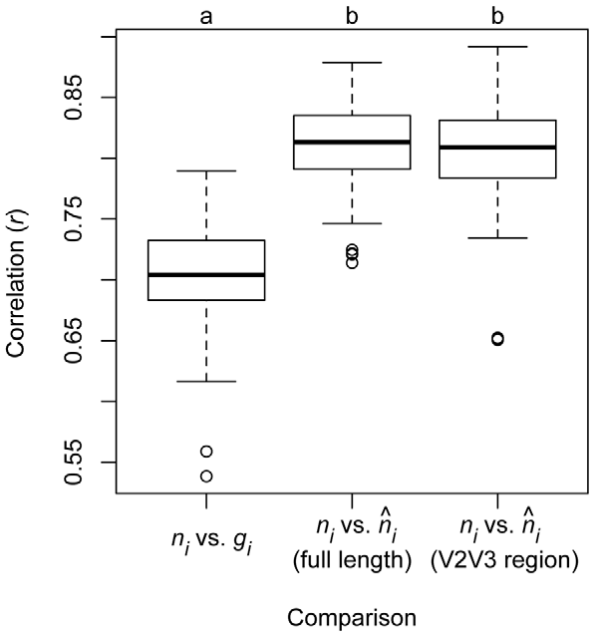
The strength of correlations between true abundance (*n_i_*) versus observed gene abundance (*g_i_*) or estimated relative abundance (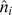) for 100 simulated communities generated by drawing 100 taxa from the 484-taxon reference phylogeny followed by estimation of the phylogenetic placement and copy number for those taxa. We simulated phylogenetic placement and copy number estimation using full-length 16S sequences and sequences trimmed to the 351 bp V2V3 hypervariable region to simulate pyrosequencing data. Letter codes at top of panel indicate simulations that differed according to a Tukey HSD test (*P*<0.05; simulations that share a letter not significantly different).

Our simulations of community OTU abundance and copy number estimation indicated that estimated organismal abundances 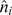 were more similar to true abundances than were gene abundances *g_i_* (Figure 6). The correlation between gene abundance and true abundance was significantly weaker (r = 0.70±0.04) than the correlation between true abundance and estimated organismal abundance 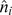 based on full-length (*r* = 0.81±0.04) and 350 bp (r = 0.80±0.04) sequences (ANOVA; *F_2,297_* = 223.5, *P*<0.001). The correlation between true copy number and estimated copy number did not significantly differ between full length versus 350 bp sequences (*r* = 0.82±0.04; ANOVA; *F_1,198_* = 0.1, *P* = 0.74).

### Case studies: effect of copy number variation on community structure in empirical data sets

**Figure 7:**
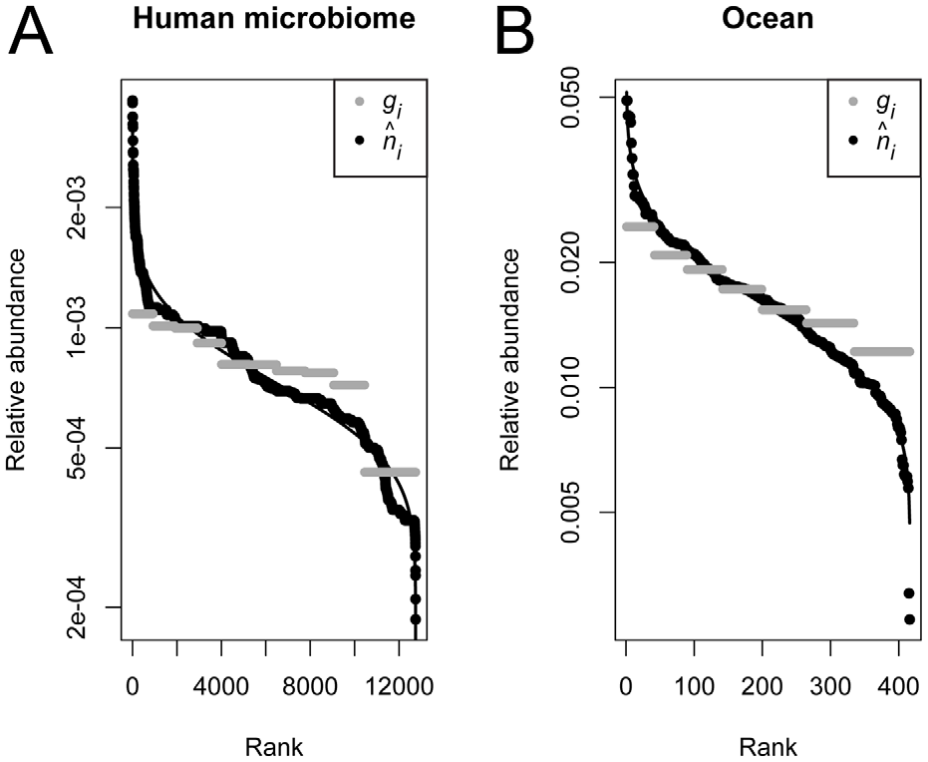
Rank-abundance distributions for two empirical microbial community data sets from (A) human skin microbiome and (B) ocean bacterial communities. Solid line indicates the expected relative abundance distribution under a lognormal distribution. Gray points are the observed relative gene abundances (*g_i_*) of sequences in each data set, and black points are the estimated relative organismal abundances (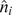).

Copy number variation can have substantial effects on inferences about numerous aspects of community diversity and structure including relative abundance distributions, the estimated abundance of different taxa, and the overall similarity of ecological communities. In both empirical data sets, rank abundance distribution plots of 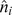 and g*_i_* revealed that failure to account for copy number variation resulted in *g_i_* underestimating the relative abundance of the most abundant taxa and overestimating the relative abundance of the rarest taxa relative to 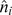 (Figure 7). The fit of empirical rank abundance distributions of 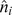 and *g_i_* to a log-normal distribution model was much better for 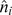 than for *g_i_* (human microbiome: AIC(g*_i_*) = −200903, AIC(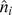) = −215791; ocean: AIC(g*_i_*) = −4573.7, AIC(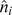) = −4808.1).

**Figure 8:**
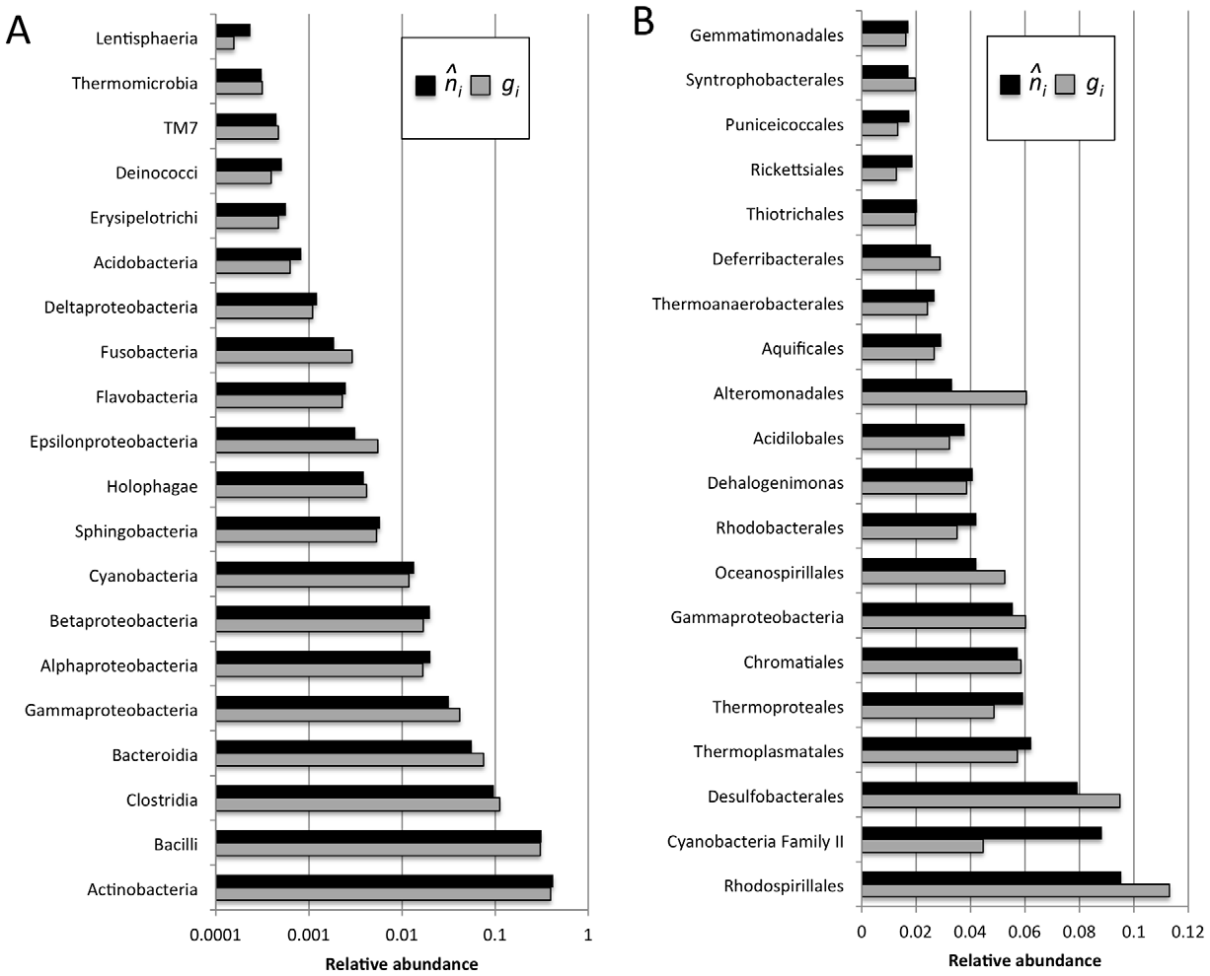
Comparison of relative abundance of the 20 most abundant taxonomic classes in (A) human microbiome and (B) ocean data sets based on observed gene abundances (*g_i_*) and estimated organismal abundances (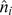).

In addition to changes in the overall shape of rank-abundance distributions, the relative abundance of several microbial taxa also changed substantially after accounting for copy number variation among taxa. In the human microbiome data set, these changes did not greatly modify the overall abundance structure of the community (Figure 8). However, in the ocean data set the relative abundance of several taxa differed greatly when based on gene versus organismal abundance estimates (Figure 8). For example, the relative abundance of sequences assigned to Cyanobacteria Family II nearly doubled and this taxonomic group went from being the ninth most abundant based on gene abundance (*g_i_* = 0.04) to the second most abundant based on estimated organismal abundance (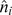 = 0.09).

**Figure 9:**
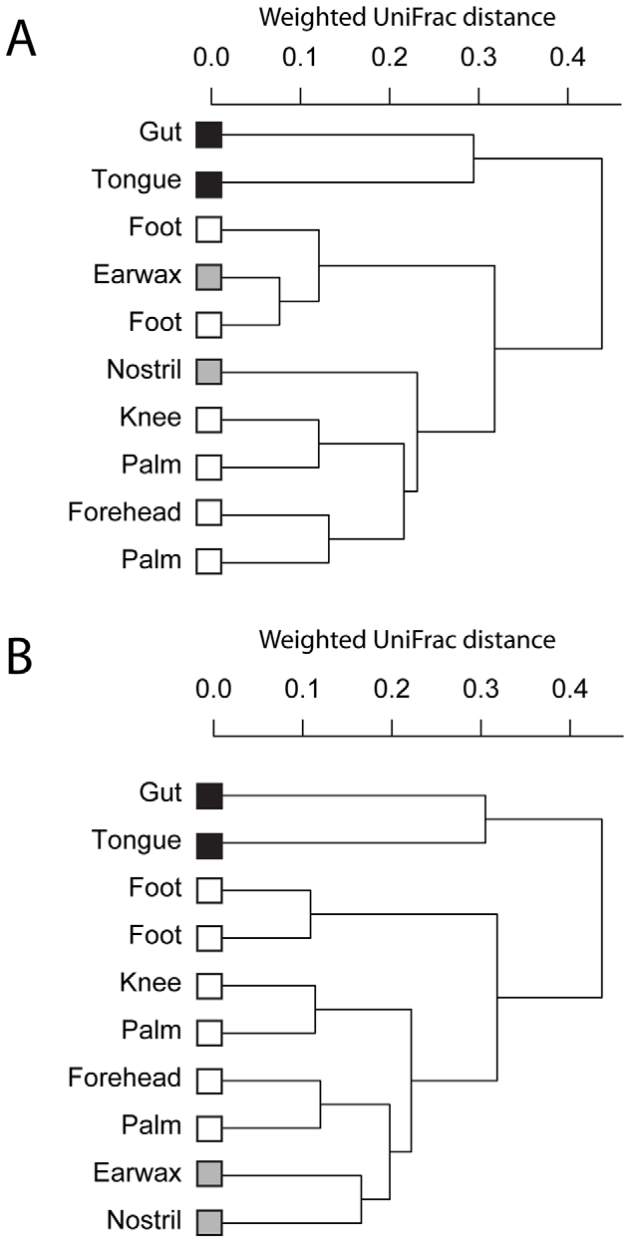
Hierarchical clustering (complete linkage) of communities from the microbiome of a human (subject F1-3 in [**13**]) based on phylogenetic similarity (weighted UniFrac distance metric) for observed relative gene abundances *g_i_* (A) and for estimated organismal relative abundances 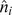 (B). Samples are shaded based on human microbiome habitat characteristics (black = gut/mouth, gray = moist skin sites, white = dry skin sites).

The use of organismal versus gene abundances did not have a major effect on the clustering of ocean communities based on their phylogenetic similarity, with samples tending to cluster together with other samples from similar depths regardless of whether *g_i_* or 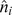 was used to calculate weighted UniFrac similarity of samples (results not shown). However, for the human microbiome data set, using *g_i_* versus 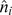 as the abundance measure changed the overall similarity of communities from different habitats as measured by hierarchical clustering of communities based on the weighted UniFrac phylogenetic distance metric (Figure 9). Based on gene abundances, microbial communities from the inner ear/earwax clustered with communities from the sole of the foot (Figure 9A), but based on estimated organismal abundance the inner ear/earwax community formed a distinct cluster with communities from the nostril, and these two communities from relatively moist skin microhabitats were compositionally distinct from all other microbial communities on drier skin sites and the gut and mouth (Figure 9B).

## Discussion

We have demonstrated how data on the sequence and abundance of 16S genes in environmental samples can be used to accurately estimate 16S gene copy number and improve estimates of organismal abundance in microbial communities. Using simulated and empirical data sets, we have shown that treating gene abundance as if it were equivalent to organismal abundance can lead to misleading inferences about microbial community structure and diversity. Our simulations indicate that genomic 16S copy number can be estimated accurately for environmental sequences through the use of phylogenetic reference data, and that failure to account for copy number variation among taxa in environmental samples can lead to the observed relative abundance of 16S sequences (*g_i_*) being weakly correlated with the true abundance of organisms in the community (*n_i_*).

Our findings have wide-ranging implications for studies treating 16S gene sequence abundances as a measure of organismal abundances in communities. In some simulations, less than 30% of the variance in true organismal abundance was explained by observed gene abundance (Figure 6). The weak correlations between observed 16S gene abundance and true organismal abundance suggest that estimation of organismal abundance from gene abundance and copy number should be a routine part of any 16S sequencing study, since it will reduce one of the numerous potential biases inherent to inferring microbial community structure from environmental sequencing data. Analyses of several empirical data sets indicated that copy number variation can affect numerous aspects of community structure that are commonly measured by studies using the 16S gene, including relative abundance distributions, estimates of the abundance of different taxa, and overall measures of community diversity and similarity.

The effects of copy number variation on community structure will not be consistent across studies, as they will depend on the relative copy number of taxa in a particular community, and on the distribution of, and relationship between, *N_i_* and *C_i_* in that community. Our simulations of gene and organismal abundance distributions, *P(G)* and *P(N)*, indicate that these distributions can have different properties. Under the simulation parameters we explored, there was a tendency for *P(G)* to have lower abundances for the rarest species and higher abundances for the most abundant species in comparison with *P(N)*. Estimates of species richness based on gene abundances were also consistently lower than estimates based on organismal abundances. These differences are likely due both to the fact that *P(G)* is a function of *P(C)* and *P(N)* (cf. Equations 2 and 3) leading to a difference in the shape of gene and organismal abundance distributions, and due to sampling depth being effectively lower for gene abundance distributions than for organismal abundance distributions for a given number of genes/individuals sampled, since multiple copies of the genes of each organism make up the pool of genes in the community. We simulated *P(N)* and *P(C)* as statistically independent distributions, but it is also possible to imagine situations where *P(N)* and *P(C)* are correlated (e.g. where abundant taxa have consistently higher or lower 16S copy number), which could further obscure relationships between gene abundance and organismal abundance.

In the abundance distributions for the empirical data sets we examined, we observed that gene abundances were generally higher for the rarest taxa and lower for the most abundant taxa compared to estimated organismal abundances, a pattern opposite that seen in our simulations. This discrepancy highlights the fact that relationships between gene and organismal abundances will vary depending on numerous factors including the distribution of organismal abundances and copy numbers as well as the relationship between organismal abundance and copy number in natural communities, and further highlights the need to estimate copy number and organismal abundance for empirical data sets.

There was not always a large effect of using gene versus organismal abundance to measure community structure in the empirical data sets we examined, but we did see major impacts on our inferences about community structure in some data sets, including changes in estimates of the identity of the common and rare taxa within communities and the similarity of communities among different habitats. If there is not a consistent difference in copy number between abundant and rare taxa, there could be little effect of adjusting relative abundance to account for copy number, but the only way to assess differences in gene versus organismal abundances for a particular community will be to estimate copy number and organismal abundance for the taxa in that community.

There is great interest in understanding the structure and dynamics of the “rare biosphere”, the rare microbial taxa whose detection in ecological communities was only possible with the advent of high-throughput sequencing technology and deep sequencing of environmental samples [45]. In our simulations and analyses of ecological communities, we found that estimates of the relative abundance of rare taxa were consistently affected by variation in copy number, likely due to the fact that the effects of copy number on detection probability and abundance estimation will be strongest for the rarest taxa in a community [46]. It will be useful to disentangle the effects of copy number variation versus ecological rarity per se on our perception of the ecology of the rare biosphere.

The phylogenetic method for copy number estimation we present in this study could be applied to predict any microbial trait for which reference sequence and trait data are available, and will help to further develop a trait-based approach to microbial ecology [47]. Numerous hypotheses about the environmental distribution of microbial traits including genomic 16S copy number have been proposed [48], [49] and it will be possible to test these hypotheses using estimation of the traits of microbial communities. This approach will complement metagenomic approaches to understanding the distribution of microbial traits and functions, since it could be applicable to phenotypic traits of microbes that cannot be directly measured from metagenomic data such as genomic copy number or ecological attributes of taxa such as growth rate or pathogenicity.

Since uncertainty in copy number estimates depends on the branch length separating environmental sequences from reference sequences, there will be greater uncertainty in estimates of copy number for sequences from poorly known and unculturable bacterial clades lacking close relatives in reference genomic data sets. However, for the empirical data sets we analyzed, the largest standard error of copy number predictions was less than one copy per sequence, even for the environmental sequences distantly related to all taxa in the reference data set. Our ability to estimate copy number accurately will be improved as the genomes of greater numbers of uncultured and rare microorganisms continue to be sequenced. The method we present in this study can be used with any set of reference sequences, and as greater numbers of genomes from uncultured and phylogenetically diverse microbes are sequenced [28], we expect that our ability to estimate copy number and abundance will become even more accurate.

Understanding patterns of organismal abundance across space, time and environments lies at the core of microbial biodiversity and biogeography research. The ability to estimate copy number and abundance for microorganisms based on environmental sequences opens the door to the application of numerous ecological methods developed for estimating taxa richness, taxa range distributions, and community similarity while taking variation in detection probability into account. Future studies utilizing the copy number and abundance estimation approach we have developed will improve our understanding of the structure and dynamics of microbial communities.

## Supporting Information

Dataset S1 Software for copy number and organismal abundance estimation.
(ZIP)

Figure S1 Histogram of genomic 16S copy number variation across the 881 bacterial genomes in the full reference data set.
(TIF)

Figure S2 Error (absolute difference between observed and predicted) and bias (difference between observed and predicted) for genomic 16S copy number predictions based on leave-one-out cross-validation for 484 bacterial taxa in pruned reference data set. Error bars indicate standard error across 100 random draws of reference taxa from the 484-taxon reference phylogeny.
(TIF)

Text S1 Effects of reference data set size on copy number estimation accuracy.
(PDF)
