# Supplementary figures and images for: Incorporating 16S Gene Copy Number Information Improves Estimates of Microbial Diversity and Abundance
Source: PLoS Comput Biol. 2012 Oct 25;8(10):e1002743. doi: 10.1371/journal.pcbi.1002743 (PMC3486904; doi:10.1371/journal.pcbi.1002743)

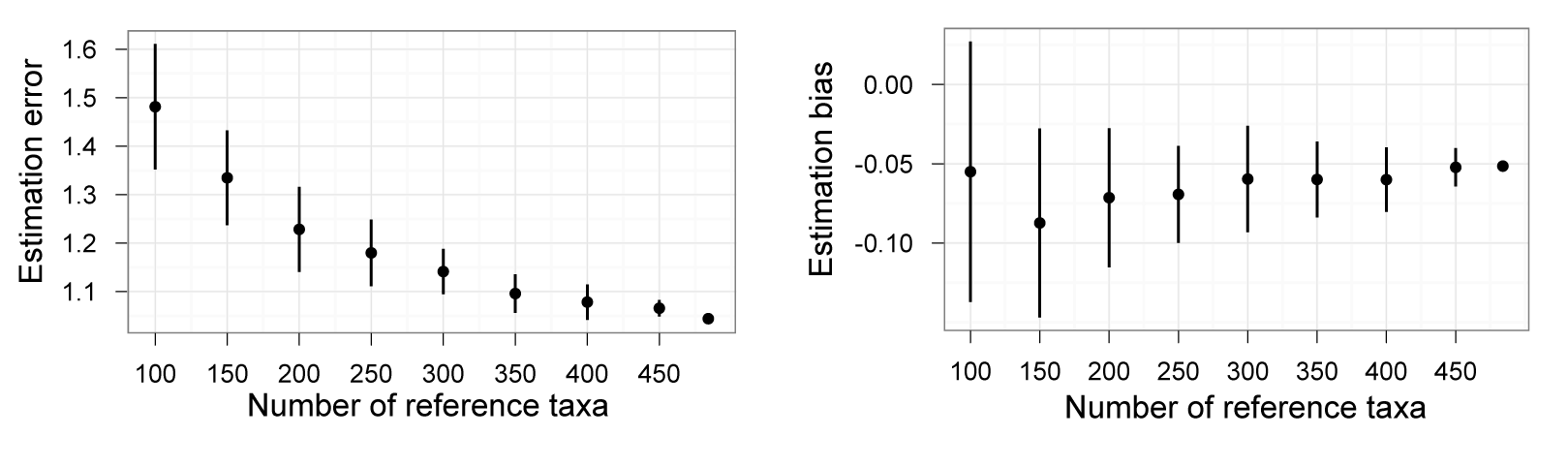

Supplement: Figure S1 — Histogram of genomic 16S copy number variation across the 881 bacterial genomes in the full reference data set. (TIF) [file pcbi.1002743.s002.tif]

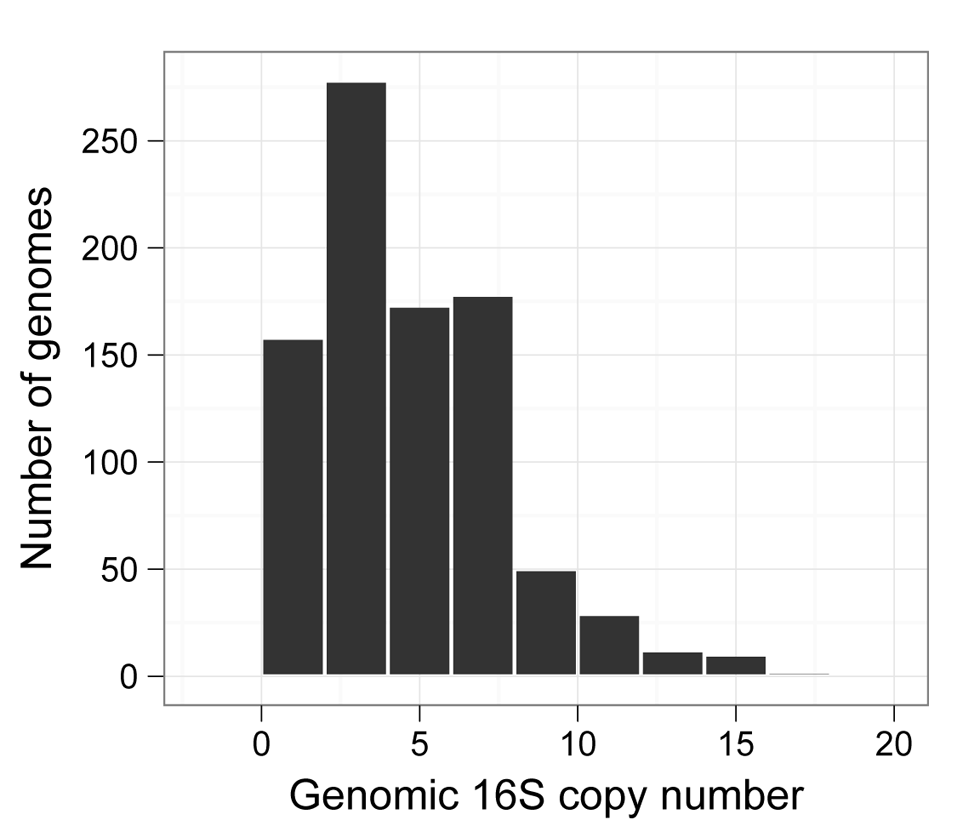

Supplement: Figure S2 — Error (absolute difference between observed and predicted) and bias (difference between observed and predicted) for genomic 16S copy number predictions based on leave-one-out cross-validation for 484 bacterial taxa in pruned reference data set. Error bars indicate standard error across 100 random draws of reference taxa from the 484-taxon reference phylogeny. (TIF) [file pcbi.1002743.s003.tif]
