# Supplementary material for: Incorporating 16S Gene Copy Number Information Improves Estimates of Microbial Diversity and Abundance
Source: PLoS Comput Biol. 2012 Oct 25;8(10):e1002743. doi: 10.1371/journal.pcbi.1002743 (PMC3486904; doi:10.1371/journal.pcbi.1002743)
Supplement: Text S1 — Effects of reference data set size on copy number estimation accuracy. (PDF) [file pcbi.1002743.s004.pdf]

Supporting Text S1.

Effects of reference data set size on copy number estimation accuracy.

We quantified the effect of changes in the size of reference data sets on uncertainty in predictions of copy number using leave-one-out cross-validation on the pruned reference phylogeny and copy number data set. We estimated prediction bias as the mean of all differences between estimated and observed copy number, and prediction error as the mean of all absolute differences between estimated and observed copy number. We evaluated the effect of changes in reference data coverage on prediction bias and error by reducing the number of reference sequences, asking at what size of reference data set accurate predictions of copy number can be made.

The leave-one-out cross-validation analysis of observed and predicted copy number for subsets of the 484 reference taxa indicated that copy number can be predicted accurately through the use of phylogenetic prediction methods, even for data sets smaller than the reference data set we employed (Supporting Figure S2). For subsets of the reference data set, bias was relatively unaffected by the number of reference taxa, with a tendency for slight under-prediction of copy number at all sample sizes, and prediction error increased for smaller numbers of reference taxa. However, even at the smallest number of reference taxa examined (100), copy number could still be predicted with a mean (± s.e.) error of 1.48 ± 0.13 copies.
